# Comparison of Blood Gas Analysis and Auto-Analyzer Results for Sodium and Potassium Levels in Elderly and Non-elderly Adult Emergency Department Patients

**DOI:** 10.7759/cureus.62225

**Published:** 2024-06-12

**Authors:** Ali Cankut Tatliparmak, Muhammed Furkan Ozden, Rohat Ak, Sarper Yılmaz

**Affiliations:** 1 Emergency Department, Uskudar University, Istanbul, TUR; 2 Emergency Department, Memorial Sisli Hospital, Istanbul, TUR; 3 Emergency Department, Arnavutkoy State Hospital, Istanbul, TUR; 4 Emergency Department, Kartal Dr. Lütfi Kırdar City Hospital, Istanbul, TUR

**Keywords:** bland-altman, sodium and potassium discrepancies, blood gas measurements, diagnostic testing, geriatric emergency medicine

## Abstract

Objectives

This study aims to evaluate the concordance between blood gas and biochemical measurement methods for sodium and potassium levels in elderly and non-elderly patients within an emergency department (ED) setting.

Methods

A retrospective method comparison study was conducted at an ED from February 1, 2023, to March 1, 2023. The study included 414 patients, categorized into "elderly" (aged 65 and above; n = 138, 33.3%) and "non-elderly" (aged 18 to 64; n = 276, 66.7%) groups. Concordance was assessed using Bland-Altman, Passing-Bablok, and Lin's concordance correlation methods.

Results

In sodium measurements, the elderly group exhibited an average bias of −1.52 mEq/L (95% confidence interval [CI] −2.12 to −0.92), with lower and upper limits of agreement (LoA) at −8.46 and 5.42 mEq/L, respectively, indicating a broader variance than non-elderly patients, who showed an average bias of −0.82 mEq/L with limits of −4.97 to 3.32 mEq/L. For potassium, the elderly group's average bias was −0.46 mEq/L (95% CI −0.36 to −0.57), with limits of agreement from −1.68 to 0.75 mEq/L, compared to non-elderly patients with a bias of −0.29 mEq/L and limits of −0.71 to 0.13 mEq/L. Furthermore, concordance correlation coefficients revealed a reduced agreement in the elderly for both sodium (*r*_ccc_ = 0.799) and potassium (*r*_ccc_ = 0.529) compared to the non-elderly cohort (sodium *r*_ccc_ = 0.821, potassium *r*_ccc_ = 0.715).

Conclusion

The study identifies significant discrepancies in sodium and potassium levels between elderly and non-elderly patients, suggesting a need for diagnostic precision. It emphasizes the importance of customizing diagnostic approaches to better serve the elderly population in EDs.

## Introduction

By 2030, it is projected that one in six individuals globally will be classified as elderly [1]. This demographic shift is expected to lead to an increase in chronic diseases associated with aging, intensifying the demand for medical services and posing significant challenges in elderly healthcare, including common comorbidities and health disparities [2]. Emergency departments (ED) serve as the primary point of access for elderly patients, who exhibit distinct utilization patterns, including higher rates of ED visits [3]. This necessitates a shift in clinical approaches and management strategies to meet the specific needs of elderly patients. Given the role of EDs as the initial contact point and the complexity involved, addressing these differences in treatment and management is paramount.

EDs are characterized by their inherent unpredictability, broad spectrum of cases, and perpetual need for rapid responses, presenting unique challenges. These challenges are particularly pronounced when dealing with elderly patients, given their physiological vulnerabilities, further underscoring the critical importance of swift and cost-effective diagnostic methods [4]. Within this context, the diagnostic tools employed in EDs exhibit a wide range. Among these diagnostic tools, blood gas analysis (BGA) plays a pivotal role in assessing both critically ill patients and those with metabolic disorders within the ED [5]. Blood gas analyzers furnish clinicians with a comprehensive array of evaluations, encompassing assessments of acid-base equilibrium, electrolyte levels, glucose, hemoglobin, and hematocrit parameters. This evolution in practice has prompted clinicians to progressively incorporate additional parameters facilitated by these analyzers into their diagnostic repertoire. It is imperative to acknowledge, however, that in scenarios necessitating dynamic monitoring for early diagnosis and treatment, certain assessments still rely on laboratory-based data as the gold standard [6].

The geriatric population experiences various factors, such as changes in fluid intake, physical limitations, polypharmacy, diminished homeostatic capacity, neuroendocrine alterations, and more [7]. These factors collectively influence their physiological responses and contribute to an increased susceptibility to issues like electrolyte imbalances [8]. In this context, BGA assumes significant importance, particularly concerning the measurement of non-respiratory parameters such as potassium, sodium, glucose, hemoglobin, and lactate, in the evaluation of elderly patients [9]. However, the current body of literature has not definitively clarified the extent to which blood gas findings align with the outcomes derived from laboratory-based biochemical analyses. The objective of this study is to evaluate the concordance between BGA and laboratory-based auto-analyzer results for sodium and potassium levels in elderly patients compared to non-elderly adults. By identifying and analyzing any significant discrepancies between these two methods across different age groups, we aim to improve diagnostic accuracy and clinical decision-making in the ED setting.

## Materials and methods

Study design

This retrospective method comparison study was carried out within the ED of Arnavutkoy State Hospital during the period from February 1, 2023, to March 1, 2023. Ethical approval was granted by the Clinical Research Ethics Committee at Haseki Training and Research Hospital prior to commencing the study (approval date: July 26, 2023; protocol number: 122-2023). All research procedures strictly adhered to the principles outlined in the Helsinki Declaration.

Selection of participants

Patients aged 18 and above who presented to the ED were considered for inclusion in this study. These individuals were categorized into two distinct groups based on their age: the "elderly," comprising patients aged 65 and above, and the "non-elderly," consisting of adult patients aged 18 to 64 [10]. The inclusion criteria encompassed patients for whom the primary emergency physician ordered BGA, potassium, and sodium tests upon their admission to the ED. Patients who had undergone cardiopulmonary resuscitation (CPR) upon admission and those who had received any medical interventions or treatments before blood sample collection were excluded from participation in the study. Additionally, patients with a time difference of more than 10 minutes between the requisition of tests were also excluded.

Outcomes

The objective of this study was to analyze the concordance between blood gas and biochemical measurement methods for sodium and potassium levels in elderly and non-elderly patients.

Data collection

Given the retrospective nature of the study, data were retrieved from the hospital's information management system (HIMS). Data were collected and filtered systematically through the HIMS, and a meticulous quality control process was undertaken by the research team to ensure data accuracy and reliability. In our institute, following collection, the blood samples were promptly transported, as per the ED protocols, to a designated room within the central laboratory, equipped with dedicated instruments for emergency use. The hospital's quality management system ensured continuous monitoring, adhering to specific turnaround time guidelines: blood gas results within 15 minutes, complete blood counts within 30 minutes, and biochemical parameters within 90 minutes. BGAs were conducted using the RapidLab 1265 analyzer, while sodium and potassium parameters were analyzed using the Cobas C501 analyzers. These instruments were routinely calibrated at the recommended intervals by the manufacturer and subjected to regular scrutiny by the hospital's quality assurance unit.

Analysis

The statistical analysis of the study was conducted using Statistical Package for Social Sciences v29 (IBM Corp., Chicago, IL, USA) and MedCalc Statistical Software (version 20.104). The sample size was determined to be a minimum of 50 patients in accordance with the requirements for method comparison studies [11]. The normal distribution of the data was assessed using histograms. Continuous variables were described as medians (IQR 25th-75th). The comparison between independent groups was performed using the Mann-Whitney U test. Categorical variable comparisons between the two groups employed the chi-square test and, where applicable, the Fisher's exact test. To facilitate the analysis of concordance between different measurement methods, a combined approach previously employed was utilized, incorporating the Bland-Altman, Passing-Bablok, and Lin’s concordance correlation methods [12]. The clinical agreement limits were defined based on values established by Clinical Laboratory Improvement Amendments (CLIA) [13]. Lin's concordance correlation coefficient (*r*_ccc_) was employed for correlation analysis, with Pearson's *ρ* and bias-corrected factor (*b*_c_) values used to assess the strength of agreement between measurements [14]. The Passing-Bablok analysis included intercept, slope, and residual standard deviation (RSD). A slope value encompassing 1 within a 95% confidence interval (CI) indicates no proportional bias, whereas an intercept containing 0 within a 95% CI indicates no constant bias [15]. The presence of systematic bias was determined using the Bland-Altman analysis, where a 0 value within the 95% CI for bias indicates no systematic bias. Prior to conducting the Bland-Altman analysis, we ensured that the difference between the two measurements followed a normal distribution. Limits of agreement (LoA) were defined as the mean difference of measurements ± 1.96 standard deviation of differences [16]. All statistical tests employed a two-tailed approach, and a significance threshold of p < 0.05 was deemed the criterion for statistical significance.

## Results

A total of 414 patients were enrolled in the study, and their demographic and laboratory characteristics are presented in Table 1.

**Table 1:** Basic demographic and laboratory values in elderly and non-elderly groups Data are presented as median (interquartile range) or number (percentage).

Parameter	Non-elderly (n=276, 66.7%)	Elderly (n=138, 33.3%)	p-value
Age (in years)	40 (27–54)	74 (68–81)	<0.001
Sex (female)	142 (51.4%)	74 (53.6%)	0.676
Blood gas			
Potassium (mEq/L)	3.92 (3.65–4.21)	4.09 (3.84–4.41)	<0.001
Sodium (mEq/L)	137.7 (135.83–139.1)	137.25 (134.65–140.3)	0.863
pH	7.4 (7.38–7.44)	7.4 (7.36–7.43)	0.193
Biochemistry			
Potassium (mEq/L)	4.2 (3.94–4.48)	4.7 (4.19–5.03)	<0.001
Sodium (mEq/L)	138.5 (136–140)	138 (136–142)	0.241

When categorizing patients by age, 66.7% (n = 276) of them belonged to the “non-elderly” group, while the remaining 33.3% (n = 138) constituted the “elderly” group. Agreement analyses were conducted using the Bland-Altman analysis (Table 2), the Passing-Bablok analysis (Table 3), and Lin's concordance correlation analysis (Table 4).

**Table 2:** Mean bias and agreement limits of parameters according to Bland-Altman analysis in elderly and non-elderly populations LoA: level of agreement; CI: confidence interval

Population	Parameter	p-value	Mean bias (95% CI)	Lower LoA (95% CI)	Upper LoA (95% CI)
Non-elderly	Potassium (mEq/L)	<0.001	−0.29 (−0.32 to −0.26)	−0.71 (−0.75 to −0.67)	0.13 (0.09–0.17)
	Sodium (mEq/L)	<0.001	−0.82 (−1.08 to −0.57)	−4.97 (-5.4 to −4.54)	3.32 (2.9–3.75)
Elderly	Potassium (mEq/L)	<0.001	−0.46 (−0.36 to −0.57)	−1.68 (−1.85 to −1.5)	0.75 (0.57– 0.93)
	Sodium (mEq/L)	<0.001	−1.52 (−2.12 to −0.92)	−8.46 (−9.49 to −7.44)	5.42 (4.4–6.45)

**Table 3:** Results of Passing-Bablok analysis in elderly and non-elderly populations RSD: residual standard deviation; CI: confidence interval

Population	Parameter	Intercept (95% CI)	Slope (95% CI)	RSD (+1.96 RSD)
Non-elderly	Potassium (mEq/L)	−0.26 (−0.49 to −0.02)	0.98 (0.93–1.04)	0.15 (−0.3 to 0.3)
	Sodium (mEq/L)	12.8 (2.9–23.93)	0.9 (0.82–0.97)	1.48 (−2.91 to 2.91)
Elderly	Potassium (mEq/L)	0.16 (−0.28 to 0.66)	0.84 (0.74–0.93)	0.42 (−0.83 to 0.83)
	Sodium (mEq/L)	16.92 (−1.4 to 30.95)	0.87 (0.77–1)	2.38 (−4.67 to 4.67)

**Table 4:** Results of Lin’s concordance correlation coefficient analysis in elderly and non-elderly populations CI: confidence interval; *r*_ccc_: Lin's concordance correlation coefficient

Population	Parameter	Lin's *r*_ccc_ (95% CI)	Precision (p)	Accuracy (Cb)
Non-elderly	Potassium (mEq/L)	0.715 (0.666–0.757)	0.877	0.815
	Sodium (mEq/L)	0.821 (0.780–0.855)	0.844	0.973
Elderly	Potassium (mEq/L)	0.529 (0.426–0.619)	0.664	0.780
	Sodium (mEq/L)	0.799 (0.736–0.849)	0.840	0.952

**Figure 1:**
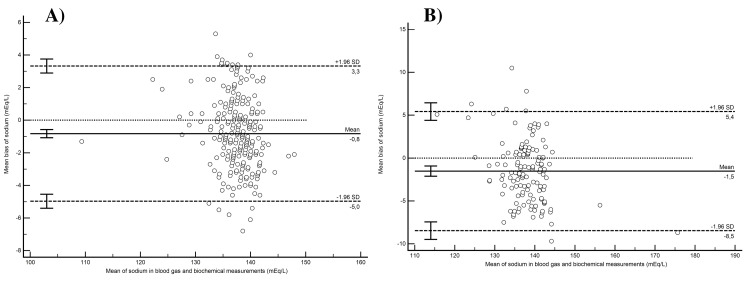
Bland-Altman analysis of concordance between blood gas and biochemical measurement methods for sodium in non-elderly and elderly patients (A) Mean bias and limits of agreement for sodium measurements according to Bland-Altman analysis in the non-elderly group. (B) Mean bias and limits of agreement for sodium measurements according to Bland-Altman analysis in the elderly group.

In the sodium measurement conformity comparisons within the non-elderly group, an average bias of −0.82 (95% CI: −1.08 to −0.57) mEq/L was measured, with the lower LoA at −4.97 (95% CI: −5.4 to −4.54) mEq/L and the upper LoA at 3.32 (95% CI: 2.9-3.75) mEq/L. Constant bias (intercept 12.8 [95% CI: 2.9-23.93]) and proportional bias (slope 0.9 [95% CI: 0.82 to 0.97]) were observed between the measurement methods. Concordance correlation analysis indicated weak agreement among the measurement methods (*r*_ccc_ = 0.821 [95% CI: 0.780-0.855], *ρ* = 0.844, *b*_c_ = 0.973). For sodium measurements conducted in the elderly, the average bias between the methods was −1.52 (95% CI: −2.12 to −0.92) mEq/L, with the lower LoA at −8.46 (95% CI −9.49 to −7.44) mEq/L and the upper LoA at 5.42 (95% CI 4.4 to 6.45) mEq/L. No constant bias (intercept: 16.92 [95% CI: −1.4 to 30.95]) or proportional bias (slope: 0.87 [95% CI: 0.77-1]) was observed between the measurement methods. Concordance correlation analysis indicated weak agreement among the measurement methods (*r*_ccc_ = 0.799 [95% CI: 0.736-0.849], *ρ* = 0.840, *b*_c_ = 0.952). Figure 1 provides a graphical illustration of the Bland-Altman analysis, showcasing the concordance between blood gas and biochemical measurement methods for sodium.

**Figure 2:**
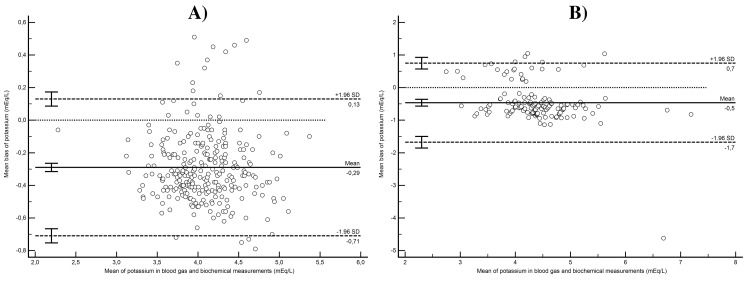
Bland-Altman analysis of concordance between blood gas and biochemical measurement methods for potassium in non-elderly and elderly patients (A) Mean bias and limits of agreement for potassium measurements according to Bland-Altman analysis in the non-elderly group. (B) Mean bias and limits of agreement for potassium measurements according to Bland-Altman analysis in the elderly group.

In the potassium agreement analysis for the non-elderly group, an average bias of −0.29 (95% CI: −0.32 to −0.26) mEq/L was determined, with the LoA at −0.71 (95% CI: −0.75 to −0.67) mEq/L and the upper LoA at 0.13 (95% CI: 0.09-0.17) mEq/L. In the concordance analysis, constant bias was detected (intercept: −0.26 [95% CI: −0.49 to −0.02]), while no proportional bias was observed (slope: 0.98 [95% CI: 0.93-1.04]). Concordance correlation analysis indicated weak agreement (*r*_ccc_ 0.715 [95% CI: 0.666-0.757], *ρ* = 0.877, *b*_c_ = 0.815). For the analysis conducted in the elderly, an average bias of −0.46 (95% CI: −0.36 to −0.57) mEq/L was observed, with the lower LoA at −1.68 (95% CI: −1.85 to −1.5) mEq/L and the upper LoA at 0.75 (95% CI: 0.57-0.93) mEq/L. While no constant bias was detected between the measurement methods (intercept: 0.16 [95% CI: −0.28 to 0.66]), proportional bias was observed (slope: 0.84 [95% CI: 0.74-0.93]). Concordance correlation analysis indicated weak agreement (*r*_ccc_ = 0.529 [95% CI: 0.426-0.619], *ρ* = 0.664, *b*_c_ = 0.780). Figure 2 visually presents the Bland-Altman analysis, illustrating the concordance between blood gas and biochemical measurement methods for potassium in both non-elderly and elderly patients.

## Discussion

BGA is crucial in emergency medicine for its quick, comprehensive insights. Despite being a standard diagnostic tool, studies reveal inconsistencies in results between blood gas and other devices, even with identical analyzers [17,18]. These variations suggest influences beyond static principles, including patient-specific factors. Literature review shows general agreement between blood gas and biochemical measurements, but discrepancies in electrolytes have been noted, especially in unique populations like newborns [19]. This study focuses on the elderly, underrepresented in research, highlighting the risk of diagnostic and therapeutic delays in an aging society. Elderly patients, although fewer, often require blood gas analysis, emphasizing the need for accurate diagnostics in this group [20]. The study aims to address the gap in understanding blood gas measurement reliability in elderly patients, considering their distinct physiological changes and the impact on emergency care outcomes.

Diagnosing sodium electrolyte disorders is challenging due to non-specific symptoms and a lack of distinct EKG findings, necessitating reliable laboratory tests [21,22]. These disorders, common in the elderly and often linked to chronic conditions or medication use, require careful monitoring due to their significant impact on morbidity and mortality [23,24]. Our study compared sodium measurements in non-elderly and elderly patients using two methods, finding similar median values but a greater average bias in the elderly (−0.82 vs. −1.52 mEq/L), yet within CLIA acceptable limits [13]. Despite no statistically significant difference in Lin's concordance analysis, the correlation was higher in elderly patients, suggesting no compromised concordance between blood gas and biochemistry methods for sodium measurements in this group. Previous studies by Xie et al. and Triplett et al. support our findings of strong concordance, despite observed average biases [17,25]. These results underscore the importance of precise evaluation of blood gas results in emergency medicine for both elderly and non-elderly patients, especially when dealing with life-threatening conditions.

Abnormal serum potassium levels, encompassing both hyperkalemia and hypokalemia, are consequential due to their critical role in cellular physiology and the potential for causing electrophysiological disturbances in the cardiovascular system. It has been documented that patients presenting with potassium anomalies in the ED are predominantly older and exhibit a heightened mortality risk [26]. This accentuates the importance of meticulous potassium level management, especially in elderly patients, given the narrow confidence intervals and methodological limitations in measurement. Our study's findings indicated that the median potassium values in the elderly group were significantly higher than those in the non-elderly group across both measurement methodologies, aligning with prior research [27]. However, it is concerning to note that agreement analyses revealed values falling beneath the 0.5 mEq/L CLIA-recommended threshold for both age groups [13]. In the elderly cohort, the LoA displayed critical differences, such as −1.68 mEq/L for potassium, highlighting a significant finding. While non-elderly patients showed no fixed or proportional bias, a notable proportional bias was observed in elderly patients, suggesting a pronounced discrepancy in elevated potassium levels. This is corroborated by a study by Açıkgöz et al., which found significant disparities in potassium measurements between blood gas and biochemical methods in cases of moderate to severe hyperkalemia, mirroring our findings [28]. The marked average biases in our study, compared to other literature, underscore the complexity of potassium measurement and its implications [17,25]. Furthermore, the study by Johnston et al. underscores that measurement discrepancies in hyperkalemia and cardiac arrest scenarios reflect not only the limitations of the devices but also the dynamic nature of patient-specific factors [29]. Moreover, the comparative analysis of concordance correlation coefficients between the elderly and non-elderly groups in our study (0.529 vs. 0.715) reveals a diminished agreement in potassium measurement methods among the elderly. The observed discrepancies between BGA and auto-analyzer results for potassium levels suggest that age-related factors dynamically influence measurement accuracy. While the exact reasons for these changes are not fully understood, it is likely that both physiological and pathological changes associated with aging, such as alterations in renal function, body composition, hormonal regulation, and the presence of chronic diseases, contribute to this variability. Consequently, managing potassium levels in clinical practice, especially among the elderly, requires a nuanced understanding of these age-related challenges. Clinicians should adopt an adaptive approach to measurement that accounts for the potential variability introduced by aging, thereby ensuring more accurate diagnosis and treatment.

Limitations

This study is subject to several limitations that warrant consideration. First, our study cohort comprises patients whose inclusion was contingent upon their primary emergency physicians' decisions to request a comprehensive panel of tests, including blood gas, glucose, sodium, and potassium. It is possible that the reasons for requesting these tests may vary among these patients, which could introduce variations in the data. As such, generalizing our findings to a broader population should be done cautiously. Second, it is important to note that this study was conducted exclusively at a single hospital, and therefore, the results are contingent upon the practices and protocols specific to this institution. Variations in procedures and equipment at different healthcare facilities may yield divergent results. Third, owing to the retrospective nature of our study, the accuracy and completeness of the data hinge on the information sourced from the hospital's information management system. The potential for data entry errors or omissions must be considered when interpreting the study's results. Additionally, while the sample sizes meet the minimum requirements for method comparison studies, the difference in group sizes (138 elderly vs. 276 non-elderly adults) should be noted, and future studies should aim for larger and more balanced cohorts.

## Conclusions

Our comparative analysis between blood gas and biochemical measurements in elderly versus non-elderly patients showed that the elderly have greater discrepancies in sodium and potassium levels, indicating potential risks to current diagnostic accuracy. These findings suggest that ED protocols need refinement to better accommodate the physiological complexities of the elderly. By analyzing the variance in test results between these two age groups, our study highlights the necessity of adjusting diagnostic approaches to improve care for the elderly, focusing on ensuring that treatment decisions are informed by precise and reliable data, especially in the management of electrolyte imbalances.
